# Exploring Design Requirements for Repurposing Dental Virtual Patients From the Web to Second Life: A Focus Group Study

**DOI:** 10.2196/jmir.3343

**Published:** 2014-06-13

**Authors:** Panagiotis E Antoniou, Christina A Athanasopoulou, Eleni Dafli, Panagiotis D Bamidis

**Affiliations:** ^1^Medical SchoolFaculty of Health SciencesAritstotle University of ThessalonikiThessalonikiGreece

**Keywords:** education, medical, dental, focus groups, patient simulation, problem-based learning, video games

## Abstract

**Background:**

Since their inception, virtual patients have provided health care educators with a way to engage learners in an experience simulating the clinician’s environment without danger to learners and patients. This has led this learning modality to be accepted as an essential component of medical education. With the advent of the visually and audio-rich 3-dimensional multi-user virtual environment (MUVE), a new deployment platform has emerged for educational content. Immersive, highly interactive, multimedia-rich, MUVEs that seamlessly foster collaboration provide a new hotbed for the deployment of medical education content.

**Objective:**

This work aims to assess the suitability of the Second Life MUVE as a virtual patient deployment platform for undergraduate dental education, and to explore the requirements and specifications needed to meaningfully repurpose Web-based virtual patients in MUVEs.

**Methods:**

Through the scripting capabilities and available art assets in Second Life, we repurposed an existing Web-based periodontology virtual patient into Second Life. Through a series of point-and-click interactions and multiple-choice queries, the user experienced a specific periodontology case and was asked to provide the optimal responses for each of the challenges of the case. A focus group of 9 undergraduate dentistry students experienced both the Web-based and the Second Life version of this virtual patient. The group convened 3 times and discussed relevant issues such as the group’s computer literacy, the assessment of Second Life as a virtual patient deployment platform, and compared the Web-based and MUVE-deployed virtual patients.

**Results:**

A comparison between the Web-based and the Second Life virtual patient revealed the inherent advantages of the more experiential and immersive Second Life virtual environment. However, several challenges for the successful repurposing of virtual patients from the Web to the MUVE were identified. The identified challenges for repurposing of Web virtual patients to the MUVE platform from the focus group study were (1) increased case complexity to facilitate the user’s gaming preconception in a MUVE, (2) necessity to decrease textual narration and provide the pertinent information in a more immersive sensory way, and (3) requirement to allow the user to actuate the solutions of problems instead of describing them through narration.

**Conclusions:**

For a successful systematic repurposing effort of virtual patients to MUVEs such as Second Life, the best practices of experiential and immersive game design should be organically incorporated in the repurposing workflow (automated or not). These findings are pivotal in an era in which open educational content is transferred to and shared among users, learners, and educators of various open repositories/environments.

## Introduction

### Virtual Patients

From as early as the 1980s, the amount of available medical information has doubled every couple of years [1]. This has led to the implementation of computer-assisted learning in many aspects of health care education. From simple indexed medical data repositories to full-fledged online virtual medical education institutions [2], nothing showed more promise for health care professionals’ education than virtual patient educational cases. Virtual patients have been defined as “interactive computer simulations of real-life clinical scenarios for the purpose of medical training, education, or assessment” by the MedBiquitous Consortium for the development of health care technology standards [3]. The need for streamlining the virtual patient creation process became apparent and standardization solutions were offered [4] with a formal MedBiquitous virtual patient initiative for the smooth exchange of virtual patients across systems and institutions being finalized in a formal International Standard form since 2010 [5,6]. Virtual patients, with current Web-based rapid development and deployment cycles, can be ubiquitously present in the curriculum (lectures, exams, project-problem-based learning, synchronous or asynchronous e-learning sessions) [7]. This proliferation of virtual patients has led to attempts of highly specialized, context-specific virtual patient design models for catering to specific medical specialties [8], or the use of virtual environments’ immersiveness by deploying virtual patients in environments such as Second Life [9].

### Serious Games and Multi-User Virtual Environments

The evolution of computer games and massively multiplayer online role-playing games (MMORPGs), specifically, has led to an interesting spin-off, the multi-user virtual environment (MUVE). A MUVE has been defined as a synchronous, persistent network of people, represented as avatars, facilitated by networked computers [10]. The characteristics of this definition also describe the inherent advantages of this platform for educational purposes. The synchronous and persistent networking of users seamlessly facilitates collaboration between them in pursuit of a common goal, be it the defeat of a powerful monster in a game environment or the treatment of a virtual patient in a health care educational environment. Additionally, the avatar representation, the graphical depiction of the user in a human likeness of her/his choice, implicitly facilitates the immersion of the user in the virtual environment. This immersion enables the user to participate in the online events with an invaluable experiential intensity [11].

Second Life is one of the oldest MUVEs. As such, it is quite mature in resources and stability. It has spun off an open source, multiplatform, multi-user, 3-dimensional (3D) application server, OpenSim [12], from which many more contemporary MUVE grids have spawned. In fact, grids such as Kitely [13] or Avination [14] are already mature MUVEs with graphics creation and scripting capabilities. However, Second Life, as the pioneer in the market, is the most recognizable of the MUVEs. The multitude of health care resources, studies, and locations in it [15-52] creates a precedent of a de facto recognizable platform for deploying educational content for health care. Although we aim to leverage new MUVEs for the deployment of future cases, in our first repurposing effort in the MUVE space we chose the most recognizable, standardized, and stable—albeit dated—platform, which is Second Life. It provides a persistent online environment where users connect to it from their computers utilizing a viewer program through which they interact with the environment (Linden Labs) provides a default viewer, but also has provided the means for the community to develop its own versions of it with different capabilities). The user controls her/his avatar by mouse and keyboard; communication modes include text chat, an email-like instant message system, and built-in voice chat with distance-based volume control. Users in this MUVE can purchase land and use it to create their own objects consisting of primitive objects (prims). Applying a simple scripting language (Linden Scripting Language; LSL), these objects can be programmed to respond to environmental- or user-initiated events to create custom interactive audiovisual experiences [53].

### Health Care Content in Second Life

Despite the criticism regarding the barriers that MUVEs and Second Life impose on the educational settings [54,55], health care content is abundant in Second Life. First of all, Second Life has been used as a treatment aid in several situations in which patient immersion in a virtual environment seemed beneficial. Such situations include addiction studies [15], weight maintenance studies [16], and building sexual health awareness [17]. Several health care resources in Second Life deal with mental health care, specifically for social anxiety disorder [18,19], delineating delusional beliefs [20], or even the study of the psychodynamics of transference [21].

Regarding the topics encountered in Second Life medical education material, one can find many and diverse aspects of the medical curriculum from the foundations of it, such as anatomy [22], to specialty material, such as pediatric primary care [23], pneumology [24], cardiopulmonary resuscitation [25,26], and emergency medicine and care [27,28]. Other efforts include such diverse topics as disability health care [29], or pharmacy student training in communication skills [30] or in the general aspects of their specialty [31].

There are a significant number of pure simulations in Second Life, such as a human immunodeficiency virus (HIV) epidemic simulator [32] and a transfusion operation simulator [33], but the bulk of the focus in the Metaverse is on building awareness through serious games and experiential learning tools, both for students (eg, Ohio State Medical Center, a virtual place for educational purposes [34]) and for postgraduate continuing medical education (eg, Wiecha et al [35]).

Simulations of dangerous or potentially dangerous activities have been implemented as scenarios in Second Life [36,37]. Nursing training has a strong presence in the Second Life virtual environment. The literature is teeming with studies and reviews about Second Life and nursing education [38-43], from general facilitation of nurse education with facilitated journal clubs [44], improving interpersonal interview skills [45], and cultivating decision-making capabilities [46], to specific subjects, such as mental health nursing [47], or meta-education, such as the education of faculty about the methods of teaching nursing [48]. This could be attributed to the combination of the necessity in nursing education for hands-on experience in an environment where no human life will be put at risk in conjunction with the lack of specialized hardware and software to simulate nursing work and the ease of development of Second Life resources through a simple scripting language.

In contrast, in the field of dentistry—an equally demanding, hands-on health care profession—only a limited number of resources have been created in Second Life in the last few years [49-52], whereas there are a large number of standalone full-fledged virtual reality simulators regarding specific dentistry applications [56-59].

This is only a cursory glance of the material available in the Metaverse. For more details, the reader is directed to Kamel Boulos et al [11] and HealthCyberMap [60] for a more comprehensive catalog of medical education resources in Second Life.

Nevertheless, even from this simple excursion in the Second Life medical education space, there is a great deal of content available and a significant research interest exists for both the creation of new and the migration of existing educational content in this virtual environment.

### Educational Content Repurposing and Standards

The idea of educational content repurposing (ie, transferring and reusing resources originally created for a certain educational context into another context) has matured in the past years [61]. However, interest in that direction goes further back. As early as 2002, a standard for metadata (physical or digital information about an object) suitable to describe every learning resource was established as the learning object metadata (LOM) standard [62]. A couple of years later, with the imminent explosion of Web-based e-learning platforms, the need for reusability and interoperability of content and platforms led to the establishment of the Sharable Content Object Reference Model (SCORM), which consisted of a collection of standards and specifications aiming to facilitate standardized content packaging, delivery, and consumption of content [63]. In 2008, the MedBiquitous Consortium for health care technology standards established the Health Care LOM scheme to address the specific needs for medical education content [64]. As this infrastructure became mainstream, an effort was initiated, in the form of the mEducator project, “to critically evaluate existing standards and models in the field of e-learning in order to enable state-of-the-art medical educational content to be discovered, retrieved, shared, repurposed, and re-used across European higher academic institutions” [61]. Both Web 2.0 mash-up technologies and federated, semantic, Web-based learning content management systems were explored as possible avenues of standardizing the repurposing of medical education content [65]. In the virtual patient section of this research, a Web-based platform (Linked Labyrinth+) [66] managed to integrate the standardized repurposing metadata scheme into the already existing virtual patient metadata scheme, thereby enabling the publication of virtual patients in the semantic Web and their subsequent consumption from all compatible semantically enabled platforms [67].

### Repurposing Virtual Patients From the Web to the Multi-User Virtual Environment

The next logical step appears to be repurposing (in a standardized manner) existing virtual patients from the Web to the virtual environment. However, this is not a straightforward issue. Some years ago, inspired by a critical review of the then current state of e-learning [68], a discourse was initiated into the intricacies of the virtual environments as an educational medium and the nuances that would be required for a truly successful deployment/repurposing of Web-based content (virtual patients or otherwise) to them [69]. It was postulated that the unique features of the virtual environment (seamless collaboration, sensory immersion, etc) should lead content creators to apply different principles when authoring for the virtual environment than when authoring for the Web. In the authors’ own words: “It would be more useful to investigate the 2 modalities (3D and 2D) in this context, as different but complementary and synergistic media rather than as competing media trying to replace one another” [68,69].

Regarding the transfer of virtual patients from the Web to the Second Life MUVE, what design principles will optimally leverage the advantages of it as a virtual patient deployment platform? A multitude of virtual patient content exists in Second Life (eg, [23-30,34-37,39,46,49-52]) and there are efforts to assess the effectiveness of Second Life as a medical education platform [35], but no attempt has been made so far to document user feedback regarding the strengths and weaknesses of the repurposing process for virtual patient content from a Web-based virtual patient player to the Second Life MUVE.

### The Focus Group

In the investigation of these experiential and immediate educational modalities, the initial assessment method of choice emerges to be the focus group study. In a detailed review of the focus group methodology in medical education, Barbour [70] demonstrated the advantages of this assessment type in experiential forms prevalent in medical teaching, such as case-based and problem-based learning, and their technological evolution, the virtual patient. Fast and effective, focus group studies have the significant advantage of allowing changes to occur on the educational episode (eg, change the content of a course during a semester instead of waiting for a time consuming statistical study) far quicker than other more traditional methods, such as the survey or the personal interview, which usually require the ending of the educational effort to provide results. Additionally, focus group assessment has the distinct advantage of diluting the power imbalance between the student and the teacher, a very important fact when trying to get an immediate feel for experiential and student-centered learning modalities. Furthermore, the ability of the focus group methodology to capture the ambience and the atmosphere of a group regarding the whole of a subject matter can lead to insights that are difficult to explore through the traditional avenues of assessment [70]. Despite the significant challenges regarding correct group composition and good facilitation during focus group meetings, the significant goal that can be achieved through a focus group study if it is well designed and executed is “theoretical generalizability” [71]. In brief, focus group studies facilitate the rapid deduction of theoretical insights into a subject matter, which later can be used to both affect change in the aforementioned subject matter or to become the trigger for further quantitative studies [70].

It is the aim of this work to use a dentistry virtual patient as a pilot case for a first exploration (via a student focus group) of the design modifications that need to be implemented when repurposing virtual patient content from the traditional Web-based virtual patient player to the Second Life MUVE deployment platform.

## Methods

### Overview

**Figure 1:**
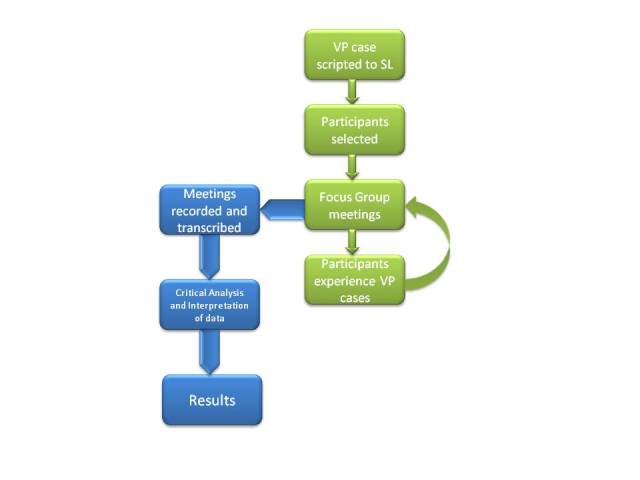
Flow diagram demonstrating this study’s overall methodology. The green area demonstrates the collection of data and the blue area demonstrates analyzing and extracting meaningful results from these data. VP: virtual patient; SL: Second Life.

A periodontology virtual patient case deployed on the Web was transferred in every detail to the Second Life MUVE. A group of dental students were asked to run both the Web-based and the Second Life cases. These students were organized into a focus group to provide their feedback regarding their experience and possible avenues for improvement. A flowchart of the process is demonstrated in Figure 1.

### Virtual Patient Case in OpenLabyrinth

The virtual patient case used for our study was a periodontology patient suffering from drug-induced gingival hyperplasia. The optimal learner course followed the correct sequence of treatment, resorting to surgery after attempting the prerequisite alternatives, while adhering to the correct procedures for the patient’s care. The case explores the knowledge of the correct surgical procedures, but does not include training in the manual techniques required in surgery.

**Figure 2:**
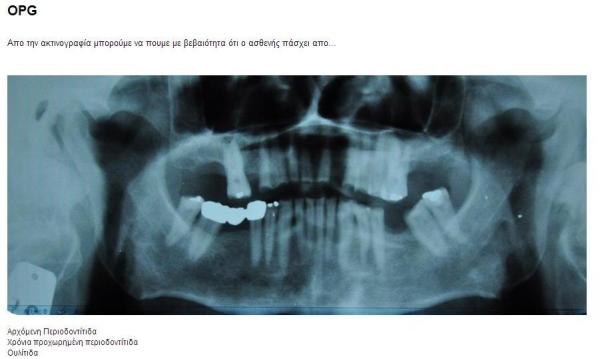
Characteristic screenshot from the OpenLabyrinth case. From the image, the learner is asked if the patient has starting periodontitis, chronic advanced periodontitis, or gingivitis.

The Web-based virtual patient was deployed using the OpenLabyrinth open source virtual patient authoring platform [72]. The case was authored as a branching scenario with multiple-choice user responses guiding the path of the case. The user interface was a standard Web browser window in which a small description of the situation was given along with any relevant images (Figure 2). The user navigated the case through multiple-choice responses available at the bottom of the Web page. A 10-minute time limit was imposed for each learner to finish the case; the case ended in a failure if the learner exceeded this time limit.

### Virtual Patient Case in Second Life

**Figure 3:**
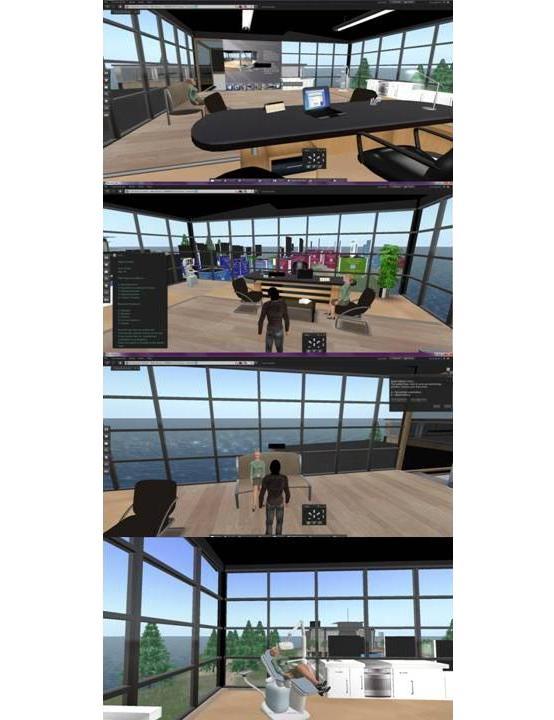
Second Life case screenshots. The user is introduced through a presentation (top image), then proceeds to the case taking cues from chat messages (second top image), and making choices through multiple-choice cards (second bottom image). The simulation involves the user interacting with the patient both in the office environment and on the dental chair (bottom image).

The case in Second Life was created in the form of a point-and-click adventure (Figure 3). The user is introduced to the case by a presentation. Then the user is guided by chat messages through the adventure and interaction with the environment through multiple-choice menu cards. The case aimed to evaluate and teach the learner, with the adventure proceeding according to her/his choices. This virtual case was designed with focus on the imparting and establishment of knowledge; therefore, there were fail states. But, most of the player’s choices were evaluated by the narrator with the option of retrying, which significantly narrowed down the end states of the adventure. This was intentional to give the player the opportunity to learn without the added frustration from unnecessarily repeating all the adventure after each failed step. Because this was a prototype virtual patient case in Second Life and because we did not have any means of assessing the time limit for negotiating the case in the virtual environment, no time limit was imposed on the users in the Second Life virtual case. Additionally, it was felt that imposing a time limit in the context of an interactive adventure that “spanned” several weeks would be detrimental to the user’s immersion in the assigned role in the case. The Second Life virtual case was developed in the privately owned island of the Lab of Medical Physics of the Medical School of Aristotle University of Thessaloniki. On that island, an indoors environment (office) of sufficient space was modified to emulate a dentist’s office. All graphical assets were either bought from other users in the Second Life Marketplace or they were modified from simple prims.

**Figure 4:**
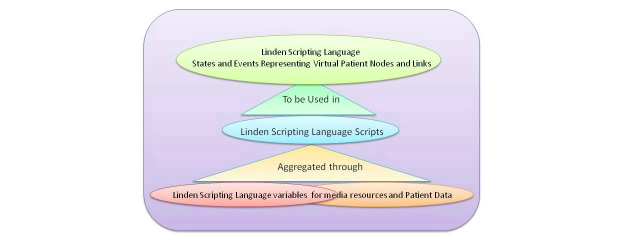
Second Life virtual patient development methodology.

All the functionality of the virtual patient simulation was coded using LSL and the necessary Web resources (eg, images) were archived for retrieval on one of the lab’s servers. LSL is an event/state-based language. The script utilizes events, such as clicking on (touching) objects, the presence (listening) of chat messages, or the creation (rezzing) of items in the environment. These events can be used as triggers within the LSL script to trigger a specific response from the script or to change the script’s state thereby enabling it to respond to a different set of events. Through these small building blocks, complex interactive behavior can be created in the simulator. Through the scripting environment, each node of the virtual patient was coded as a specific state that the case script entered when the appropriate situation occurred. The activities of the case were coded as events that triggered as the user interacted with the environment by touching pieces of it and receiving challenges in the form of multiple-choice questions. The user’s response triggered additional events that led the case to move to the next relevant state/node. Additionally, all the patient data, narrative, values, or external media references were stored in the case script as global variables. Each in-world object contained its own script that communicated with the main simulator scripts to facilitate interaction through clicking on objects. To create interactive surfaces in parts of objects, a number of invisible, interaction objects were drawn in front of the visible geometry. The transfer of the case to Second Life took approximately 10 person-hours. Our scripting methodology is demonstrated in Figure 4.

### Second Life Scripting Methodology

Technically, this approach facilitated a streamlining of the Web-based virtual patient data from the OpenLabyrinth platform. In OpenLabyrinth, a virtual patient is represented through a branching Web page tree, whereas each node is a specific Web page containing the narrative and the relevant data along with multiple buttons to allow for the different choices. Additionally, in OpenLabyrinth, all image files and other similar patient data are stored in bulk and referenced in each node as needed to facilitate reusability of assets [73].

**Figure 5:**
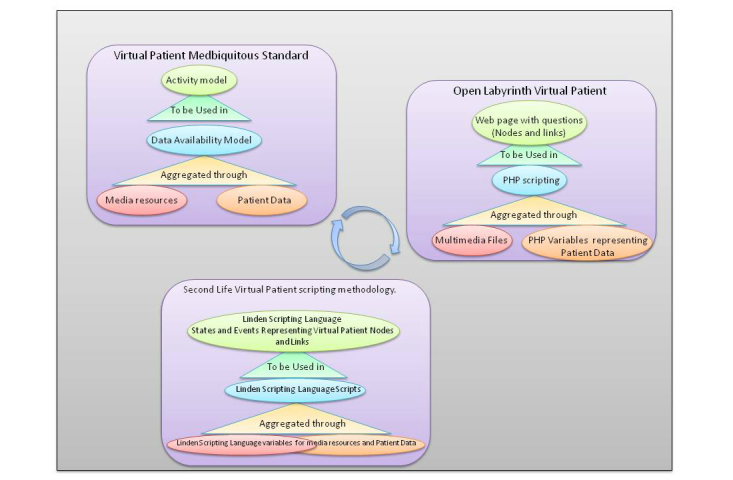
Design analogies: Second Life (SL) scripting paradigm, OpenLabyrinth deployment paradigm, MedBiquitous Virtual Patient Standard. PHP: Hypertext Preprocessor.

This design approach was used to allow the OpenLabyrinth platform to conform as a virtual patient player to the MedBiquitous Virtual Patient Standard. In that standard, media resources and patient data are aggregated to a data availability model that is used to create a structured activity model of nodes and choices that provide the desired educational outcome [3]. Mimicking, in scripting methodology, the OpenLabyrinth virtual patient deployment strategy led to analogies with the MedBiquitous Virtual Patient Standard. These design analogies between the Second Life virtual patient scripting methodology, the OpenLabyrinth virtual patient deployment platform, and the MedBiquitous Virtual Patient Standard are demonstrated in Figure 5.

### Student Group Composition and Selection Process

A group of dentistry students experienced both the Second Life adventure and the Web-based virtual case. The group consisted of 9 members, 6 male and 3 female, all dentistry students in the latter half of their studies. An open call for participants was made in an optional undergraduate course of dental informatics at the local dentistry school to participate in the project. A brief explanation of the tasks required by the participants was given and invitation was extended in a completely volunteer fashion. The focus group met 3 times in total: once before and twice after the participants had experienced the virtual case. This simple process ensured that the participants’ pool would be both thematically interested, but also engaged in providing relevant and meaningful feedback.

### Focus Group Methodology

All participants were organized into a single focus group. Feedback was received from a series of 3 meetings. Each meeting was between 25 and 45 minutes in duration and covered one of each of the major topics summarized in Textbox 1. The first group meeting took place before the participants experienced the virtual patient cases and served as an introduction and orientation session.

**Focus group meeting agenda.**

1. Computer and gaming literacy: Are you familiar with any educational computer game? If yes, which one(s)?

2. Usability of the Second Life as a virtual patient deployment platform: Have you felt that you needed personal human assistance with the interface while playing the Second Life virtual case?

3. Comparisons between the Web and Second Life deployed case: Did you feel that the tasks required of you in the Web-based/Second Life virtual case were adequately challenging? Do you think the level of difficulty differed?

In all meetings, one of the authors was appointed as a facilitator for the focus group. For the group’s meetings, a question pool was developed to initiate and facilitate the discussion. Because the goal was to allow the group members as much freedom of expression as possible, the facilitator intervened with further exploratory questions only when the discussion reached a dead end. During the discussion, the facilitator diverged many times from the question pool to explore emergent themes and opinions that came up through the discussion.

The participants were provided with the URL addresses of both the Web-deployed and the MUVE-deployed cases and were asked to complete both before the second focus group session. No specific order of case completion was asked of them. The participants used their own personal computers, laptops, or desktops to experience the 2 cases. Discussed briefly in the beginning of the second focus group session, the technical specifications of the participants’ hardware were split. Although no specifics were recorded, nongaming participants had hardware near to the medium-low performance spectrum of contemporary hardware configurations, whereas gaming enthusiasts had hardware close to the high end of the performance spectrum. One of the weaknesses of the approach that was used on exposing the users to both cases was the evoked competitive comparison of the 2 platforms in the discussions of the focus group sessions. Because all users encountered the same case across different platforms with primarily esthetic changes between them, any kind of merit or flaw of the MUVE as a virtual patient deployment platform was viewed comparatively to similar aspects of the Web-deployment platform. This comparative approach that emerged from the discussions was not suppressed by the facilitators to maintain the openness of the discussion. Instead, it was used as means for elaborating on the repurposing needs of the MUVE deployment platform.

All focus group meetings were audiotaped and transcribed with notes kept during the sessions to capture the nonverbal “mood of the moment” that could not be documented through the recordings.

The analysis of the recorded data was a process of dissecting the discussion transcripts, discovering common themes across the participants’ opinions, and noting tone, context, and mood at each stage. Then these were coded to assess possible unifying or dividing causal themes that might emerge from the discussion exploiting the immersion of the researcher to facilitate both analysis and interpretation of the data [74].

All participants were informed about the study before their appointment and a signed informed consent was obtained from each of them before the focus group meeting. The study was approved by the Bioethics Committee of the Aristotle University of Thessaloniki Medical School.

## Results

### Summary

Although all members of the focus group were at a relevantly similar stage in their dentistry studies, there was a diversification in computer literacy as shown in Table 1. Some users were significantly less experienced in computer games (reported playing games at least 8 hours per week) or professional computer use. From the first focus group session, it became apparent that all users that considered themselves familiar with gaming were intimately familiar with noncasual games, such as real-time strategy games, adventures, first-person shooters, and MMORPGs.

The interpretation of the focus group sessions led to 7 categories of issues concerning the migration and repurposing of a Web-deployed virtual patient to the Second Life MUVE. These results are summarized in Table 2.

**Table 1:** Breakdown of the group members’ computer, gaming, and educational gaming familiarity.

Computer literacy (gaming and otherwise) of focus group members	Members								
	#1	#2	#3	#4	#5	#6	#7	#8	#9
Average weekly hours of professional computer use	6	5	1	9	1	3	3	5	4
Average weekly hours of gaming	12	3	8	18	9	0	5	3	14
# of other educational computer games played	4	0	0	1	0	2	1	0	3

**Table 2:** -Focus group’s results at-a-glance.

Category	Comments
Second Life as an educational MUVE in general	Navigation in Second Life difficult to people unfamiliar with games
	Second Life graphics engine unoptimized for its capabilities
Interactivity and its educational value	Second Life more interactive than the Web-based case
	Simple Web-to-Second Life transfer is an underutilization of the MUVEs capabilities
	Users familiar with games trapped in gaming mindset implicit from the environment
	Users frustrated by disappointment of expectations because of limited interactivity
Immersiveness and its educational capacity	Users expected more immersive content from Second Life because of the nature of the platform
Clarity of educational purpose and content	In Second Life, the visual representation of the case provided significant implicit feedback and direction regarding the next step
Challenge level	Visual representation and action implied correct procedures
	Apparent decrease of challenge in Second Life
Scope of educational use	Useful as an asynchronous teaching tool
Suggestions	More complex case would better leverage Second Life’s capabilities
	Increased feedback required
	Increased interactivity and immersiveness requested
	Introduction of a human factor in the case requested

### Category 1: Second Life as an Educational Multi-User Virtual Environment

The focus group reported no issues with either registering or accessing the Web-based virtual patient. The consensus of the group was that the Web-based interface was easy to use. On the contrary, accessing the virtual patient in the Second Life proved to be a challenge for some members of the focus group. To people unfamiliar with gaming conventions, the navigation in the virtual environment seemed difficult. On the other hand, there were cases of gaming veterans reporting equal difficulties because they expected game-like challenges where none existed.

The strong points of a mature multi-user environment such as Second Life emerged through a consensus of simplicity, ease of use, and overall stability. As expected, the users most familiar with computer games had a markedly easier time interacting with the environment, another strong point of MUVEs given the pervasiveness of massive multiplayer online games (MMOG). Because this was a test case of transferring a Web-based virtual patient to the simulator, it was not a polished system, but the group considered its features as complete for the purpose it was built.

Finally, some concerns were raised about the unoptimized graphics engine; although not state of the art, it did tax the capacity of nongaming-focused computer systems:

The frame rate was very low which was tiring after some time using the system.

Yeah, and it’s odd! The graphics are really simple so I don’t understand why it was so slow.

### Category 2: Interactivity and Its Educational Value

People with a lot of experience with computer games but not with professional computer use became trapped in the gaming mindset and had preconceptions about challenges of the simulator which did not exist in it:

I flew around the building and saw the venue of the case but did not search for the entrance because I thought that there might be a puzzle to solve in order to gain access to the office.

It was clear from the instructions that the only problem to be tackled was the medical one and that no environmental barriers or hazards were present in the simulation environment. In addition, all group members (including the one offering the previous quote) were adamant that the instructions given to them were adequate and that they understood them perfectly.

Another opinion expressed, which also bears upon interactivity, was that the linear design, with primarily multiple-choice questions, constituted a disservice to the simulator’s capabilities. Participants suggested that a more nonlinear, user-driven experience would be more appropriate:

...I was forced to do what you’ve designed for me to do. I could either click to the patient to do something, or to the notepad that you had over there to take something or to the x-ray or something...I could not do anything else. I have played adventure games before, those mystery games, where I was in control of what to do. For example, I could pick up a pencil and do this, or that, or even choose not to pick up the pencil at all.

The unanimous feeling of the group was that the Second Life virtual patient case was significantly more interactive than the Web-based one:

Second Life is more immediate, you have the patient sit in the dental chair, you see him, every step, you know he will come again, it is demonstrated that what you will do requires time, it doesn’t end in a day. In the Web-based case you make choices and the patient go back and forth but it doesn’t show.

However, some members considered that the Second Life platform could facilitate a much higher level of interactivity and a much more complex virtual case:

While the environment was pleasant, the case was too linear and without enough branches to take advantage of the environment.

Moreover, the simple transfer of the case from the Web to the simulator, a fact that led to underutilization of the MUVEs capabilities, appeared to create strong feelings of frustration that were counterproductive in the negotiation of the case:

The Web case was more acceptable than Second Life because in Second Life you have more demands. In the Web it is like you are filling out a test. I had no more expectations from that...

As if it was not bad enough that it is studying, which is boring by definition (laugh), there were no good graphics to support it...forget about it! Students will want to fire up another game and play instead.

Some members of the group regarded the feedback of the case as too straightforward and provided information that they would prefer to have gathered through more steps in the case:

...It asks me if I want to take the patients history. I do and it presents me with the history record. It does not allow me to choose what to ask, how to follow up on a question, a thing that is common in an adventure where you ask a person and he replies to you and then you reply back and you lead the discussion where you want and if you lead the discussion poorly you will not get the information that you need to solve the mystery...

### Category 3: Immersiveness and Its Educational Capacity

Most of the focus group considered the Second Life virtual patient case significantly more immersive than the Web-based case:

In Second Life, there was the environment of a dental office, you imagine how it would be and it is nice to see it. Seeing the patient, it helps to create a spirit of immersion even although graphics are not state of the art.

However, the aforementioned critique regarding interactivity was transferred almost verbatim to the discussion about immersion:

...when you have to examine the patient with some tools it just lists choices a, b ,c. Why don’t you present me with the tools on a plate to look and choose a tool? After all it has the visual capability...to allow me to experience it myself...

Additionally, there were suggestions about more immersive, more stimulating feedback:

I think they could have put more medical details that would make it more realistic. I mean, I do something and it says that the patient did not respond. It would be better to tell me what was the response and if I could do something else to follow up.

### Category 4: Clarity of Educational Purpose and Content

In discussion about clarity of purpose and content, the group was unanimous that in both the Web-based and the Second Life cases there was always a clearly described medical situation that a prepared student could negotiate according to her/his knowledge without doubt about the medical problem at hand. Small differences in opinion were formed in the group regarding some issues of translating terminology in English (all students were nonnative English speakers) because of some of the members’ poor technical language skills, but these were mentioned only as minor nuisances. What was mentioned unanimously was the fact that the visual representation of the case’s progress was a kind of feedback that facilitated clarity of purpose and provided direction regarding the next step in the simulator:

*Seeing the patient in the dental chair was a significant boost to the feedback I took from the Second Life virtual patient compared to the Web-based* one*.*

Seeing the patient in the dental chair, it prompted you to think that it is time for some intervention and so it focused your mind more on the case.

### Category 5: Challenge Level

Regarding the challenge level, the group was divided with members considering the challenge level of the case adequate but a bit on the easy side, whereas others considered it adequate to challenging. An interesting point was that the perceived challenge was less in the Second Life case because of the visual props that prompted them toward the correct course of action at some points of the virtual case. Members who reported feeling challenged by the case and being facilitated by the props of Second Life did indeed finish the case on the first try, whereas the users that considered the case challenging and finished it on the second try after reaching a fail state in the first try were those who had extensive gaming experience but very little professional computer experience. Table 3 summarizes the duration of both the Web case and the Second Life one. There was a slight difference in the finish times. All participants took between 10 and 20 minutes to finish it (mean 15.1, SD 4.7 minutes); however, these times were sufficiently short that the difference can be attributed to attention differences between the participants.

**Table 3:** Comparison of time to finish the virtual cases on Second Life and the Web.

Completion time for the Second Life and Web-based virtual patient	Members (minutes)								
	#1	#2	#3	#4	#5	#6	#7	#8	#9
Time to complete the Second Life case	10	20	20	13	20	120^a^	8	15	15
Time to complete the Web case	8	5	9	7	5	3	3	10	10

^a^This time includes bibliographical referencing and study time so actual time interacting with the environment should be assessed as being much lower. This was considered an outlier and was not included in the calculation of the mean time for completing the case.

### Category 6: Scope of Educational Use

Most of the group thought that Second Life would be a very useful tool as a teaching aid, primarily used asynchronously:

...If there was such an application I would buy it for me to do it at home as training, it would be fun, to be relaxed at home and to ask the patient stuff and take responses or to do what I want in the cases...

It was suggested that it would be an invaluable tool in the training of preclinical students to familiarize them with practical matters in a safe environment before actual practice begins. Learning from errors was identified by participants as a pivotal educational/pedagogical issue, and this was properly incorporated in the Second Life MUVE:

It is good that it tells you that you made a mistake and it lets you learn from it by leading you and letting you retry. Retrying and learning from mistakes is very good for digesting the material.

### Category 7: Suggestions

Many of the suggestions of the focus group’s members were regarding the need for the cases to be more interactive and immersive:

...it would only be interesting if it had more choices, more depth of choice and interaction, less limitation in options...

I would really like to be allowed to continue down a fail path and explore the consequences of my mistake in order to understand why this was the wrong choice.

And also about feedback that would be more explanatory as opposed to just expositional of the techniques.

...it did not say why we do that, or why we will not do the other. I would like it to provide the “why,” not just the “how” of the treatment.

Exactly! It would be nice to continue, even after a failed step, in order for us to see why our choice was wrong.

From the discussion, it became clear that the group would prioritize the improvement of the case itself regarding depth and meaning of choice. A close second priority was the ability to be able to vividly see and do things in the simulator. The introduction of a human factor in the case was suggested. Some of the participants suggested multiple branching dialog options, history-taking questions, and a better exploration of the patient factor in treatment (ie, offering an effective but intrusive treatment and the patient refusing it), along with the needed management options:

...in medicine/dentistry, the patient is a human being, and, therefore, it is not a yes/no thing, ”yes he is ill/ no he is not;” there are gray situations...

...A realistic patient would have objected and asked for clarifications regarding the practices that were applied.

Another suggestion was one touching on interactivity, namely the ability of the user to utilize the simulator’s capabilities for a more hands-on experience with the practical techniques of the case:

...Surgery could be simulated, not the manual practice, but by a graphical drawing of the incisions by the player on a photo and be graded by the margin by which you were off the optimal ones.

## Discussion

### Principal Results

From the focus group discussions, some interesting insights emerged. The most straightforward was the realization that the Web-based case was a direct “by the book” implementation of this virtual patient case. The focus group’s verdict was that this was indeed a platform where cases such as the one they experienced would thrive. The case’s level of interactivity and data were appropriate for the Web. The Web platform itself was the most ubiquitous in the world; thus, this simple and easy-to-use implementation of a virtual case seems the standard by which other implementations can be assessed. Although not a direct substitute for face-to-face experiential forms of learning, the Web-deployed virtual patient is established as standard learning material [75] with literature covering detailed aspects, such as the conceptual concerns of connecting specific clinical guidelines in the design of virtual patients [76] or quality control metrics for assessing them [77]. However, repurposing from the Web to the MUVE requires much more than a simple transfer of the data across the 2 platforms. The MUVE’s increased interactivity potential and rich immersive nature supports but also requires significant changes to the content of a virtual patient package to meaningfully adapt the Web-based case to the MUVE platform. The deployment of virtual patients in a 3D MUVE is far less explored than the Web-based one where there have even been attempts to facilitate the deployment of such virtual patients directly from the staff of health care institutions, to facilitate a more rapid development of virtual patient content and increase awareness of the virtual patient educational medium [78]. First, the Second Life platform, with its 3D environment and its references to both real-world verisimilitude and increasingly pervasive MMOGs, prepares the user to expect a significantly increased level of interactivity from anything deployed in it. In fact, many users, specifically the avid gamers, expected so much more from a 3D MUVE that the simple transfer of the less interactive and nonexperiential Web-based case harmed the educational effort by frustrating them and caused aversion to the poorly implemented (for the platform’s capabilities) case.

Second, for a virtual case to be relevant in a MUVE it should have a significant amount of complexity. A basic tree structure with some short branches to a couple of fail states or to some retry nodes does indeed work on the Web where its ubiquitous pervasiveness and ease of use provides a rapid way for learners to confirm or reinforce their knowledge with no mediating learning or computational overhead. The proliferation of virtual patients as a learning resource [7] has led to exploration of their usability as assessment tools, but only in the Web-deployment platform [79,80]. Things change drastically when considering the 3D MUVE deployment platform. When a user has to invest a serious amount of time to enter a specific virtual environment with its own conventions, control schemes, and system requirements, then that user expects a rich branching case, similar to a real minigame, that will engage both sensorially and intellectually to keep the user focused and present. This kind of experience could be a knowledge confirmation tool but also a significant, even the primary, disseminator of knowledge regarding the subject matter that the case is negotiating. Additionally, interesting contemporary evolutions could be leveraged to increase interactivity and spontaneity in the users’ reactions. For example, interactions through nonscripted, natural language processing [81] with the simulator would greatly enhance the sense of presence of the user, especially if the simulator’s response came from a nonscripted artificial cognition process, or the medical challenges came not prescripted, but emerged from full-fledged vital signs simulation engines (eg, [82-84]). However, as was revealed by the discussions of the focus group, for the system to play that role, the case itself should consist of a complex tree with several branches that lead to interesting and meaningful consequences after each user choice providing an experience more akin to one that would happen in a real-world environment and less like one in a controlled test-like environment with artificially imposed barriers.

Third, in order for a Second Life deployed virtual case to be an effective learning tool, the quantity of interactivity should be great and so should its quality. The sensory capacity for immersion in a MUVE is both a blessing and a curse. A blessing for the user in a fully developed case who can meaningfully interact with the environment moving through and freely manipulating the objects present there and using manual practices that she will be called to apply in a real-world environment. For example, in the 3D virtual environment it seemed odd to the focus group members to be asked to identify medical tools by name when they could be modeled in front of a tray and picked up and used by the user. However, this is also the curse for the author and developer of the case in the MUVE. One must resort as little as possible to narration through text and utilize the sensory immersive potential of the MUVE by creating rich and immersive audiovisual content to present the user with all the pertinent information. In every other case, she risks alienating the user base who, trained from the all-pervasive MMORPGs, have very strongly established preconceptions of what to expect from any environment that is presented in 3D and where they interact through their avatar. Almost all the members of our focus group praised the interactivity of the MUVE compared to the Web, but they found it heavily lacking considering the potential that the MUVE platform has and the expectations that this created for them. It is not unrelated that all the suggestions offered for improving the case were toward that goal either through more realistic manipulation of the in-world objects or the more hands-on manipulation in the manual procedures applied to the case (eg, surgery).

### Context

This work was triggered by the repurposing efforts applied to the mEducator program [61]. In that program funded by the European Commission, the main goal was the identification of the prerequisites, the development of infrastructures, and the piloting of a streamlined process for repurposing medical educational content across European academic institutions [61]. For this reason, a specific architecture and a dedicated schema was developed for federating and semantically enriching content across learning content management systems (LCMS) [67]. The overarching purpose was to make educational content discoverable and context-naïve to facilitate its reusability and repurposing for different educational goals and across different educational environments [61].

A significant part of the aforementioned project was the semantic enrichment of Web-based virtual patients for them to be discoverable and reusable across different contexts and purposes [85]. However, virtual patients can be repurposed pedagogically, but also technologically. Repurposing virtual patients to Second Life could provide a new platform for implementing the results of the Linked Labyrinth+ mEducator project [85,67].

The Linked Labyrinth+ semantic enrichment of virtual cases was a first important step in making the virtual patient content discoverable and repurposable across learning objectives and academic institutions [85,67]. However, establishing sound repurposing principles to the Second Life platform would be the important missing link that would allow the use of existing content as the basis for creating custom virtual patients from existing material, or even incorporating procedural methods [86] of game design to create dynamic virtual patient content. At this point, the Second Life MUVE is rather dated. Because of its age, unoptimized graphics engine, and cumbersome user interface (UI), the experience is rather difficult at times. However, these drawbacks, along with the maturity of the platform and the multitude of existing health care content, were the reasons that led to the choice of this platform for exploring repurposing of virtual patients from the Web to the MUVE. By conducting our test in a rather cumbersome and dated platform and subsequently deducing repurposing guidelines in this “difficult” environment, it is ensured that future deployments in more current MUVE platforms will provide an even smoother experience. Moving beyond the limitations, the repurposing guidelines that emerge from this study could facilitate the use of even more open-ended virtual patients and eventually lead to procedural virtual patients. Additionally, such semantic enrichment in the context of a highly interactive 3D virtual environment could facilitate the game feedback in the form of artificial intelligence (AI) avatars that can provide meaningful challenge or assistance to a user by tapping into resources linked through semantic enrichment [87-89]. That kind of sophisticated user interaction and automated content creation in 3D environments would greatly enhance both the utility and the impact of the MUVE-deployed virtual patient. However exploring such options requires a clear vision of what works and what does not in repurposing virtual patients from the Web to the MUVE.

Efforts for content generation in 3D environments are not novel. Especially in the field of cultural heritage, there are several efforts for migrating real-world cultural content (eg, monuments, depictions of works of art) to 3D environments (eg, [90,91]). There are even successful attempts at utilizing semantic enrichment of such content to automate the transfer from the real world to the virtual [92]. By embedding metadata from wikis and established semantic namespaces along with geospace information, there have been successful attempts to provide real-time automated updating of 3D environments to real-world cultural heritage sites [93].

Thus, an effort to put together the aforementioned 2 axes of research, the virtual patient repurposing from the Web to the 3D virtual environment and the automation by semantification of content generated in 3D virtual environments, one very important prerequisite must be met. That is the identification of the guidelines and best practices regarding the pedagogically correct repurposing of the virtual patient content from the Web to a 3D virtual environment.

This makes the current study unique, original, and valuable. Before engaging the academic teams and structures (eg, Health Sciences Education Office) in such an endeavor, one needs to investigate the suitability of the decision for undergraduate (dental) education. It is certain that the incorporation of virtual patients in medical curricula is not a novelty; there are studies of their impact and effectiveness as course materials in general medical training [94] and in dentistry [73]. However, the transition from the Web to the virtual environment is not a pedagogically straightforward one. To explore the necessities of the MUVE platform, user feedback is required.

By transferring the virtual patient from the Web to the MUVE verbatim, we have provided a single-user learning experience; that is, we have not facilitated the collaboration of multiple users for resolving the case’s challenges. The advantages of collaborative learning in medical education are well documented [95,96]; however, at this point we were focused on uncovering the differences between deployment platforms and the details of exploiting the different deployment media than to explore the multi-user dynamics of a collaborative virtual patient educational episode. It is one of the future goals of this line of research to investigate the optimal multi-user modes of interaction in a MUVE-deployed virtual patient.

This study is a first overview of the challenges that must be overcome in repurposing virtual patients to a 3D MUVE. It consists of only 1 focus group, which is not enough to provide a detailed relief of the requirements for repurposing in the MUVE. However, this was a conscious choice. This first attempt in repurposing virtual patients across platforms could only be explored in broad strokes when dealing with simple transfer between platforms. To be able to explore more details in true repurposing and no transference, a need exists to deconstruct the virtual patient down to its learning objectives and to its expected teaching outcomes to be able to repurpose either to specific topics or to specific outcomes. As mentioned in the literature [69], the 2 media should not be considered as competitive, but as complementary of one another. Thus, a repurposing effort should focus in the strong points of each platform and reinforce the teaching outcomes that can be conveyed in the most impactful way by the specific platform. For example, in a Web-based patient, the theoretical breadth of knowledge in periodontology is the area in which the platform thrives, whereas in a 3D MUVE-based patient, manual practice on incision techniques is where its graphics-rich environment can really help the learner acquire a different and complementary skill set to what the Web-based virtual patient can offer. Further qualitative study (eg, with additional focus groups) can come only after the redesigning of the MUVE-deployed virtual patient. However, to successfully achieve this task, there must be adherence to both the guidelines that emerged from the present study, but also from the aforementioned conceptual considerations. Only after such an effort has been the subject of rigorous qualitative study can a formal quantitative assessment be attempted. It becomes clear that this study, with only 1 focus group should be considered the required first step in meaningfully utilizing the MUVE as a virtual patient deployment and repurposing platform beyond the mere transference of resources.

Students, as the immediate consumers of this educational content, are the best group to provide feedback. Additionally, student input was important for discovering feedback from users familiar with the gaming culture in general. Their opinion should be considered more “expert” regarding gaming than, for example, senior faculty members. For these reasons, the authors engaged into a straightforward but rather strict process to facilitate such an exploration in this focus group study. The participants of this study were all volunteers from a relevant optional course of dental informatics in the undergraduate curriculum of a dentistry school. The fact that the student pool from which the participants’ were chosen was that of a relevant optional course ensured that only those actively interested in the subject were chosen for the study. Additionally, the voluntary character of the participation meant that even those interested in the subject, only students with active commitment for engaging with the subject would participate. These 2 factors ensured that the participants’ feedback in the focus group discussions would be both on topic and thorough.

Focus group studies can uncover subtle context, whereas survey or interview studies are best suited at quantifying established truths [70]. Our goal to explore the guidelines for the pedagogically successful repurposing of Web-deployed virtual patients to the 3D MUVE platform seemed best suited for the focus group approach. The differences of the 2 deployment platforms are subtle. Both are computer based, both have been used for game deployment in a recreational context, and both have also been used as learning facilitators. Exploring the best practices for platform repurposing in the deployment of the virtual patients requires the investigation of the user experience not only by concrete metrics of user satisfaction and system usability, but also by assessing contextually the user experience. Subtleties such as the user extrapolating from previous experiences in 3D MUVEs through serious or even recreational gaming need to be established first before being quantified.

The goal of this study was not to quantify the usability or the satisfaction of a user base regarding an information technology (IT) system in medical education, but to establish qualitative guidelines that will evolve to best practices through further study.

### Conclusions and Direction for Future Work

These insights lead to some interesting conclusions regarding the design requirements in the effort of repurposing Web-based virtual cases for MUVEs:

1. Show, don’t tell. When repurposing an existing virtual case to a MUVE, text narrative should be kept to a minimum. Instead, audiovisual assets should replace the narration to immerse the user into the narration and provide an increased sense of presence into the case.
2. Call for action, don’t call for answers. The nature of the MUVE is such that many of the challenges presented during a case can be simulated by avatar actions. When the opportunity appears then provisions should be made, even if that means additional development overhead, for the user to be able to actuate the requested solution instead of just choosing it from a multiple-choice questionnaire.
3. Consequences, not barriers. This is probably useful in all virtual case deployment platforms, but its effects are outlined with stark clarity in a MUVE because of the immediacy with which the user encounters all enforced barriers and artificial preventions of her actions. Instead, all actions of the user should provide useful feedback and lead to plausible evolution of the case instead of abrupt fail states or returns to previous choices.

These are straightforward and simple principles, but it is important to be clear that they are not optional embellishments. In the continuing effort for a systematic repurposing of Web-based virtual cases to MUVEs, the aforementioned required design practices that emerged from this focus group study are not just going to be adhered to as rough guidelines, but incorporated in the core of the workflow (automated or not) of the repurposing effort.

These guidelines should be seen as first steps in a wider context. Current trends point to open education as a method for acquiring skills and knowledge on demand. Internet technologies facilitate that kind of student-directed learning in 2 significant ways [97]. One axis of Internet technologies concerns the remote use of expensive equipment through online time-sharing and collaboration with established professionals in relevant fields, for example, by initiating a telescope observation with the help of experts through a network of remote observatories [98]. The other axis concerns the availability of specific knowledge online and on demand for “learn-at-your-own-pace” episodes [97]. This and social learning (ie, learning by asking questions and doing things relevant to the field [99]) are a perfect match for a virtual environment that contains meaningful interaction with realistic challenges. Finding the correct parameters for streamlining virtual patient repurposing from the Web to Second Life becomes more than just an interesting research niche. Instead, it is important for meaningfully combining the impact of the immersive 3D MUVE experience with the mature and growing virtual patient effort. This combination could lead to new aspects of open social learning [97] by facilitating the mass migration of the growing virtual patient content in a form that each and every learner can absorb at her own pace, but also can engage in such an experiential way as to be able to affect that “learning to be” [97] part that is missing from the Web-based virtual patients. For this future goal, the guidelines that have emerged from this work are an important first step.

## Abbreviations

LCMS: Learning Content Management System
LOM: Learning Object Metadata
LSL: Linden Scripting Language
MMOG: massive multiplayer online game
MMORPG: massive multiplayer online role-playing game
MUVE: multi-user virtual environment
SCORM: Sharable Content Object Reference Model
